# *OntoTrader*: An Ontological Web Trading Agent Approach for Environmental Information Retrieval

**DOI:** 10.1155/2014/560296

**Published:** 2014-04-01

**Authors:** Luis Iribarne, Nicolás Padilla, Rosa Ayala, José A. Asensio, Javier Criado

**Affiliations:** Applied Computing Group, Department of Informatics, University of Almeria, 04120 Almería, Spain

## Abstract

Modern *Web-based Information Systems* (WIS) are becoming increasingly necessary to provide support for users who are in different places with different types of information, by facilitating their access to the information, decision making, workgroups, and so forth. Design of these systems requires the use of standardized methods and techniques that enable a common vocabulary to be defined to represent the underlying knowledge. Thus, mediation elements such as *traders* enrich the interoperability of web components in open distributed systems. These traders must operate with other *third-party* traders and/or agents in the system, which must also use a common vocabulary for communication between them. This paper presents the *OntoTrader* architecture, an *Ontological Web Trading* agent based on the OMG ODP trading standard. It also presents the ontology needed by some system agents to communicate with the trading agent and the behavioral framework for the SOLERES *OntoTrader* agent, an *Environmental Management Information System* (EMIS). This framework implements a “Query-Searching/Recovering-Response” information retrieval model using a trading service, SPARQL notation, and the JADE platform. The paper also presents reflection, delegation and, federation mediation models and describes formalization, an experimental testing environment in three scenarios, and a tool which allows our proposal to be evaluated and validated.

## 1. Introduction

In a more open world, information systems must be flexible and readily adaptable, extendable, accessible, and operable by different people or groups of people who are in different places and have different types of information, facilitating access to information by decision-makers, workgroups, and so forth (*convergent systems*). This involves the use of rules and standards for their construction and real-time operation, interaction, and interconnection. In this kind of systems, system “agents” (e.g., web components, subsystems, and humans) working in the same* ambient* (computing space), or even other* third-party* “agents”, interact. System convergence is possible through three basic parameters: (a) autonomy and intelligence (*software agents*), (b) a common vocabulary for all convergent systems (*ontologies*), and (c) trading between subsystems, necessary to enable, coordinate, translate, and maintain this common vocabulary (*traders*). The most recent* Web Information Systems* (WIS) have been developed under open and distributed paradigms using rules and standards for the construction and operation of real time interaction and interconnection [1]. WIS are intended for the new era of information systems in web environments, due not only to the growing popularity of the web technology, but also to the roles of web technology in modern information systems. The major features of web information systems are web semantics, XML technologies, web mining and querying, and information extraction [2].

*Environmental Management Information Systems* (EMIS) [3] are a type of WIS. Experimentation with WIS technological advances in fields like medicine, biology, and especially* environment* has allowed real convergent systems to develop over time. EMIS are social and technical systems with a variety of final users and actors (i.e., politicians, technicians, and administrators) who cooperate with each other and interact with the system for decision making, problems solving, and so forth. An EMIS uses normally knowledge bases distributed in space and time. Not only is this information used by human actors in the system but also coordinates web software (and autonomous) components requiring a common vocabulary (e.g., ontologies). An example of web-based EMIS is the SOLERES system, a spatiotemporal environmental management system based on neural networks, agents, and software components [4].

An EMIS is an* Information System* supporting environmental management. EMIS are a special case of* Geographic Information Systems* (GIS), though the granularity of environmental information is more specific (e.g., satellite and ecological information). Furthermore, EMIS use a more advanced, more specific technology than geographic information systems; for instance, we use neural networks, multiagents systems, and cellular automata features.

Two basic roles interact in WIS: the* Human* and the* Computer*. Due to the nature of these systems (open and distributed), people organized in workgroups for decision making may be found in different places and be arranged by their profile (e.g., administrators, politicians, technicians, and users). This sort of interaction (*Human-to-Human*, HH) requires unified protocol and communication policies for all parts of the system. People and groups of people also interact through the system by using* user interfaces* (UI), which are generally well suited to their needs [5]. This type of interaction (*Human-to-Computer*, HC) also needs to establish communication protocols among users, profiles, groups, and user interface agents. Finally, these user interface agents act as mediators for people interacting with other system agents. This interaction (*Computer-to-Computer*, CC) requires communication protocol, commonly known as choreography (or orchestration). All three of these types of interaction protocol (HH, HC, and CC) software have to know the knowledge semantics that manages each part of the system (i.e., agents).

Mediators (commonly known as “traders”) enrich the* interoperability* of web components working in a WIS approach [6, 7]. These traders have to operate with other third-party traders and/or agents in the system, which must also use a common vocabulary to allow communication between them. The design of these components and the interaction protocols (mentioned above) must use standardized methods and techniques that allow a common vocabulary representing the underlying knowledge to be defined. Ontologies are used as a means to this goal [8].

On the other hand, popularity of the WIS has led to an increasing volume of information available in information systems.Users depend on the web to meet their information needs through search engines, portals, digital libraries, and other information retrieval systems [9, 10]. However, information overload has led to a situation where users are swamped with too much information and have to sift through the material in search of relevant content. To address these problems, a variety of techniques, inherent in information searching, drawing from the fields of information retrieval, information filtering, human-computer interaction, and the study of information search behavior have been adopted in the WIS. Information retrieval refers to techniques that assist users in meeting their information needs [11].

The main WISinformation retrieval mechanism is the traditional* Query-Searching/Recovering-Response* (QS/RR). This mechanism is based on the traditional client/server model. On one hand, the term “Query” refers to the whole process of creating and formulating the client's question. The term “Searching” refers to the process of locating the repositories where the information is found, and the term “Recovering” refers to the process of locating, identifying, and selecting the data from the data sources (repositories, data storage, or databases, regardless of the model). Finally, the term “Response” refers to the whole process of formulation, preparation, and creation of the response by the server to the client. The “Query-Searching” pair is a process that goes from the client to the server. The “Recovering-Response” pair goes from the server to the client.

UDDI (*Universal Description Discovery and Integration*) specification and WSDL (*Web-Services Definition Language*) for SOA (SOA,  http://www.oasis-open.org/) (*Service Oriented Architecture*) are based on theseclient/server implementations for web systems. Nevertheless, these techniques allow agents to respect a* subscribe/publish/response* model and a* QS/RR *information retrieval approach for locating WSDL documents (i.e., XML specifications of web-services) and connecting web services in WIS, but not for different types of information (non-WDSL information). Traders are another solution for open and distributed systems [12] that extend the OMA (OMA,  http://www.omg.org/) (*Object Management Architecture*) ORB (*Object-Request Broker*) mechanism. Although traders are traditionally used as* middleware* for object interoperability, they can easily be adapted for interoperability of information and functionality. In respect to this, the use of functional ontologies in our work is a good solution for adapting traders to information retrieval in WIS. User information retrieval is an emerging area and a promising avenue for the design and implementation of a new generation of information retrieval systems, especially for new web-based EMIS due to the particularity and complexity of the information and users (politicians, technicians, and administrators) [13].

In a sense, the SOLERES system (a spatiotemporal environmental management system based on neural networks, agents, and software components), our EMIS model, follows a WIS approach [14–17]. In this paper, we propose the* Ontological Web Trader* (*OntoTrader*) as a mechanism for solving the complexity of information retrieval in the EMIS by means of a trading model for WIS guided and managed by ontologies. This kind of web service is a user-information search service based on a web QS/RR model. Therefore,* OntoTrader* is a new  information retrieval mechanism that implements a QS/RR model and uses the SPARQL query language and the OWL ontology description language to operate. This service is based on a user request action that identifies the agents involved and their communication protocols. In our system, the ontologies are used in two different contexts: (a) they represent the application domain information itself and (b) the services that some agents request from others during their interaction. Although a trader agent has five interfaces (i.e.,* Lookup*,* Register*,* Link*,* Proxy,* and* Admin*), this paper discusses only the behavioral and data ontology design features of the Lookup trader interface, which is used for searching and recovering user information in a QS/RR model. All research work presented here is part of a complete design strategy for* Ontology-Driven Software Engineering* (ODSE) that we are developing in SOLERES.

The remainder of the paper is organized as follows.  Section 2 reviews some EMIS approaches and compares how ontologies, agents, and trading features are used.  Section 3 presents the SOLERES system as a running case study.  Section 4 defines* OntoTrader*, a WIS information retrieval mechanism. In this part, we describe the Lookup interface ontology and the metadata template that the trader manages (i.e., an OWL metadata repository of environmental information) in OWL and formalize them in UML.  Section 5 presents* OntoTrader* formalization. Sections 6 and 7 describe some implementation and experimental scenario details and some evaluation and validation discussions, respectively. We end with some conclusions and prospects for future work in Section 8.

## 2. EMIS Technology

The progress of the new technologies in web-based information systems is obvious. In a more* open world*, information systems must be flexible and readily adapted, extendable, accessible and operable by different people or groups of people (convergent systems). The convergence of these systems may appear over time with the business needs of the system. For instance, a decision-making system in medicine, originally made up of two subsystems, may increase over time by connecting to other systems in networks which require/provide contents/services to the system service as a whole. The convergence of these systems is possible due to three basic parameters: (a) autonomy and intelligence, (b) a common vocabulary for all convergent systems, and (c) mediation between subsystems, necessary to enable, coordinate, translate, and maintain this common vocabulary. In the first parameter, software agent and multiagent system properties encapsulate traditional concepts of web components (or web services) with repositories of knowledge-based rules that work autonomously (endowing it with a certain degree of intelligence). These rules can be made and applied to agent groups that work in the same communication and behavior patterns (choreography). Ontologies are a good mechanism for establishing this common vocabulary necessary in convergent systems.

*Semantic web* is a good example of this. It standardizes the criteria, languages, mechanisms, methodologies, and platforms in universal web data semantics. Finally, trading services are a good device for communication-coordination (interoperability) between WIS subsystems as, for example, UDDI and WSDL in SOA. EMIS is an example of WIS developed during recent years. Experimentation with technological advances in WIS in fields like medicine, biology, and especially environment has allowed real convergent systems to gradually emerge. In the following sections, we analyze some relevant EMIS found in the literature and see how agents, ontologies, and trading services are applied, ending with a case study, the SOLERES system architecture, as an example of an environmental information system developed by the Applied Computing Group (ACG) at the University of Almeria, Spain [4].

### 2.1. Agent and Multiagent Systems in EMIS

Software agents are applied in different contexts in information systems. They are applied in four basic ways (their hybrids) in Web-based Environmental Information Systems (our main line of research).

- In* information management*, agents can be used for searching for information (such as a database); they can filter results, recall them, organize, or even automatically distribute them.
- In* control* and* supervision* processes (monitoring), an agent can monitor a particular element or activity and respond to any event that may occur, for example, in transport system modeling and control or in industrial processes.
- In cooperative work, group applications (*Computer Supported Cooperative Work*, CSCW), where, due to their nature, agents can support the information flow necessary for the activity and interaction of group members.
- As* personal assistants*, an agent can only easily represent a user if he/she knows his/her preferences beforehand and can act according to those preferences. For example, in electronic commerce applications, the commercial transactions require access to many resources in real time, and this task can be performed by one or more agents on behalf of the user.

We used these four contexts to design and implement the QS/RR model for a web-based trading agent (*OntoTrader*), Section 4. For an MAS design, not only the properties mentioned above must be borne in mind, but also the choice of a clear, precise methodology and a development platform. There are currently over a hundred software products for agent-based application design, which are mainly used in academic and commercial environments. Some examples are AdventNet Agent Toolkit, Agent Builder, AgentTcl, AgentTalk, AgentTool, AgentWare, Cable, Emorphia, FIPA-OS, Grasshopper, Impact, JADE, MAGE, MASS, Microsoft Agent, SiWalk, Soar, and so forth. We used JADE (JADE,  http://jade.tilab.com/) (*Java Agent Development Framework*) to implement the SOLERES multiagent system because it simplifies the implementation of multiagent systems through middleware and provides a tool-set as support for depuration and implementation of open, distributed, and heterogeneous information systems.

Agent-based environmental systems are grouped into environmental information management, assistance in decision-making for environmental problems, and environmental system and process simulation. Due to our special interest in EMIS designed with agent technology, we exhaustively reviewed the most important systems.  Table 1 shows a summary of the EMIS architectures studied. We have included the following relevant system metrics used in our web trading agent proposal, including SOLERES, in the table for comparison: (a) use of trading mechanisms, (b) use of modeling and designing ontologies, and (c) use of some type of user or interface agent. We also studied the technology and the main application domain metrics. The EMIS architectures studied were InfoSleuth [18], EDEN-IW [19], NZDIS [20], FSEP [21], MAGIC [22], DIAMOND [23], and BUSTER [24].

### 2.2. Ontology Applications in EMIS

Ontologies were designed to be used in applications that need to manage information semantics. In general, ontologies not only describe spatial data, for instance, more easily understood by computers in encoded semantics, but also integrate other EMIS data (i.e., geographical) from different sources and different ways of reasoning.

There are several languages available for modeling knowledge domains [25], in particular, DAM-OIL [26] and OWL [27]. Web Ontology Language (OWL), the most recent and widely used language at present, was designed to be used in applications that need to test the content of the information instead of just representing it. Such content can either be new or related to others. An ontology can therefore use terms that are included in other ontologies and change them, creating an open, distributed system.

In the literature, ontologies have been used to represent environmental knowledge. In [28], the authors present an environmental decision-support system called OntoWEDSS for wastewater management. In this system, an ontology is used to provide a common vocabulary for modeling the wastewater treatment and an explicit conceptualization that describes data semantics. Another example may be found in [29], an air quality monitoring system, which uses an ontology to define messages and communications concisely and unambiguously. In [30], the authors present Ecolingua, an EngMath family ontology for representing quantitative ecological data. These examples show the use of ontologies to build models that describe the entities in the given domain and characterize the relationships and constraints associated with them.

In [31], the authors present an ontology for representing geographic data and related functions. To meet the need for an interoperable GIS, in [27] the authors propose a Geo-ontology model design to integrate geographic information. We have also explored an ontological application in the field of geographic information retrieval [32]. A different use appears in [6], where an OWL extension has new primitives for modeling spatial location and spatial relationships with a geographic ontology.

Extensions of existing ontologies have also appeared in this knowledge domain. In [33], the authors propose a geographic ontology based on Geographic Markup Language (GML) [34], and the OWL-S profile is extended to geographic profiles. Another case is an extension of the NASA Semantic Web for Earth and Environmental Terminology (SWEET) ontologies that includes part of the hydrogeology domain [35].

### 2.3. Trading in Open Distributed Systems

The Reference Model of Open Distributed Processing (RM-ODP) is a model jointly developed by the International Standard Organisation (ISO) and the International Telecommunication Union (ITU-T). This model defends the transparent use of services distributed in platforms and heterogeneous networks and dynamic location of these services. The* trading function* is one of the 24 functions of ISO/ITU-T ODP model [12]. This speciation was adopted by the Object Management Group (OMG) which called it CosTrading for the CORBA services trading service.

**Figure 1:**
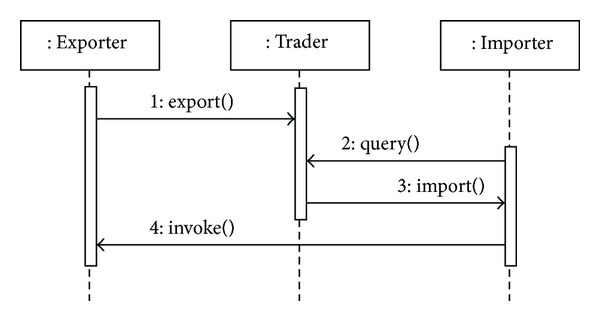
Roles of the ODP trader (*Adapted from ISO RM-ODP*).

From the viewpoint of object-oriented programming (OOP), a trading function (trader) is a software object which serves as an intermediary between objects that provide certain capacities (services) and objects that require dynamic use of these capacities. From the ODP perspective, those objects providing capacities to other objects are called* exporters* and those requiring capacities from the system objects are called* importers* (Figure 1).

A trading object uses five interfaces to interact with client objects (importer and exporter): Register, Lookup, Link, Proxy, and Admin. The Lookup interface allows clients to ask the trader about the services stored in the trading service. With the Register interface, clients can export services offered in the trader. The Link interface allows the trader to be connected to other traders (i.e., trading federation). This enables the system administrator to connect his trader with other well-known traders and propagate requests in a network. The trader can also send the request to connected traders and locate new offers with the same searching conditions imposed on the target trader. The Admin interface allows administrators to configure the trader (e.g., searching policies, ordering policies, and numbers of federated traders allowed). The Proxy interface is used to enable* legacy system* properties in federated trader systems.

## 3. A Case Study: The SOLERES System

**Figure 2:**
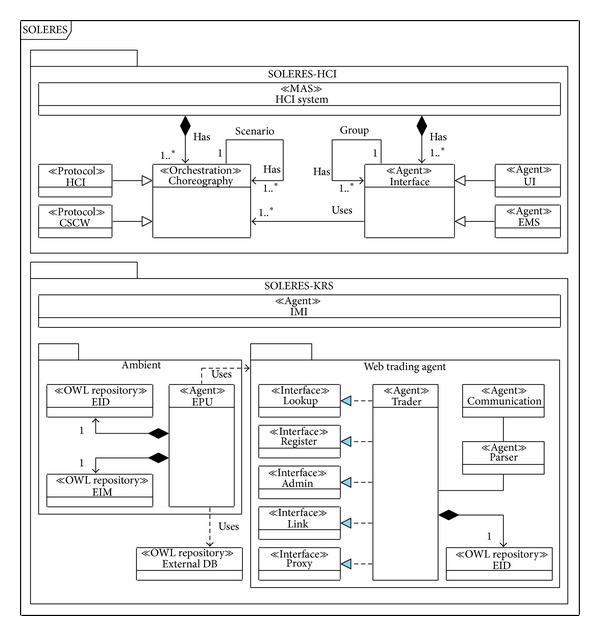
SOLERES architecture.

This section presents the main SOLERES system architecture, a spatiotemporal information system for environmental management (an example of EMIS). This system is supported by the application, integration, and development (extension) of multidisciplinary studies in satellite imaging, neural networks, cooperative systems based on multiagent architectures and intelligent agents, and software systems with commercial components. The general idea of the system is a framework for integrating the disciplines above for “environmental information” as the application domain, specifically ecology and landscape connectivity. The system has two main subsystems, SOLERES-HCI and SOLERES-KRS.  Figure 2 shows the general system architecture.

SOLERES-HCI is the framework specialized in human-computer interaction. This level of the information system is defined by means of the* Computer Supported Cooperative Work* (CSCW) paradigm and implemented using innovative intelligent agent technology and multiagent architectures. The system is designed to be used for cooperative environmental decision-making tasks by different people (systems users) arranged in different organization models (i.e., depending on their hierarchy or profile). There may be politicians, technicians, or administrators, among others, who need to interact with each other and with the system.

A user of the cooperative system has an intelligent agent (Interface agent in Figure 2), which operates two ways: (a) by managing user interface presentation and interaction (UI agent) and (b) by managing the environmental queries (EMIS agent). The UI agent mediates between the user and other users in the system (using other UI agents). The EMIS agent refers to a virtual consultant or virtual supervisor who cooperates with other agents within a previously established multiagent architecture. Both agents (i.e., UI and EMIS agents) follow protocol models for orchestration of the cooperative system, cooperation among agents. UI agents use HCI choreography (protocol) and EMIS agents use CSCW choreography.

On the other hand, SOLERES-KRS is used to manage environmental information. The  IMI agent is like a gateway between the user interface and the rest of the modules and is responsible for the management of user demands.

Given the magnitude of the information available in the information system, and that this information may be provided by different sources, at different times or even by different people, the environmental information (i.e., the knowledge) can be distributed, consulted, and geographically located in different ambients (i.e., locations, containers, nodes, or domains) called Environmental Process Units (EPU). Thus, the system is formed by a cooperative group of knowledge-based EPUs. These groups operate separately by using an intelligent agent to find better solutions (queries on ecological maps).

We accomplished the distributed cooperation of these EPUs by developing a web trading agent (*OntoTrader*) based on the ODP trader specification [12] and extended to agent behavior. Our trading agent mediates between HCI requests and EPU services. EPUs manage two local repositories of environmental information. One of the repositories contains metadata on the information in the domain itself (i.e., basically information related to ecological classifications and satellite images), called Environmental Information Map (EIM) data or EIM documents. This information is extracted from outside databases (External_DB repository in Figure 2). The EIM documents are specified by an ontology in OWL [15, 16] (<<OWLrepository>>). These EIM documents are the first level of information in the KRS subsystem.

The second repository contains metadata called* environmental information metadata* (EID), or EID documents. These documents contain the most important EIM metadata that could be used in an information retrieval service and, furthermore, incorporate other new metadata necessary for agent management itself. To a certain extent, an EID document represents a “template” with the basic metadata from the EIM document data. The EID documents have also been specified by an ontology to accomplish open distributed system requirements. EID documents represent the second level of information in the KRS subsystem. Each EPU keeps its own EID document (or sets of documents) locally and also registers them with the* OntoTrader* (the web trading agent). This way, the* OntoTrader* has an overall repository of all the EID documents from all EPUs in an ambient and can thereby offer an information search service, as described further below.

The trading service implemented and the requirements that it must fulfill to be considered open and distributed are described below. Afterwards, the structure and ontology used by the software agent responsible for this service (the trader agent) are explained.

## 4. Ontological Web Trading (*OntoTrader*)

Trading is a well-known concept for searching and locating services. In a trader-based architecture, a client component that requires a particular service can query a matchmaking agent (i.e., the trader) for references to available components that provide the kind of service required. We based our work on the traditional functionality of a trader, but with adapting it to knowledge-based agents instead of objects (or software components). But first, let us see the requirements a trader should have.

### 4.1. Requirements for Open Distributed (Web) Trading

Although the trading services could be compared to web search engines, in reality, web searches are more structured. In a trading service, the matching heuristics need to model the vocabulary, the distance functions, and the equivalence classes in a specific domain. For cooperation among web traders, trading services using different strategies should be able to federate. For example, a* repository-based federation strategy* makes it possible for different trading services to read and write in the same repository at the same time, each unaware of the presence of the others in the federation, and thereby allowing the approach to be scalable. On the other hand, the traditional “direct” federation strategy requires a trading service to communicate directly with the trading services with which it is federated. Although this federation-based scheme is very safe and effective, communication overloads increase with the number of federated trading services.

Current information searching and selection (recovery) use “hard” matchmaking operations which are too restrictive, and “soft” matching criteria are often necessary. In trading services, especially in independently extendable open systems (e.g., the Internet), it must be decided whether “soft” or “hard” matching is required. A trading service should thus allow users to be able to specify heuristic or metric functions in the search, particularly in cases where matches are “soft.” For instance, the trading service could return results ordered by a search criterion or discard some search sequences in the repositories where they are carried out.

Furthermore, after processing, a trading service should respond to a user-query request (through a SOLERES-HCI interface agent) with a result. This result, found after a* request-response* action, can refer either to a list of results that meet the restrictions imposed on the query or to a “failure” notice if the search could not find a solution. If a “failure” notice is returned, the trading service should also be able to demand that the query request must necessarily be satisfied; otherwise, the query request is stored, and the response is delayed. This* request-response* behaviour is called* store and forward* query. Finally, a trading service should also allow query requests to be delegated to other (known) traders if they cannot be satisfied by the current trading service.

Now that the demanded requirements of the trading service have been identified, the service support structure can be described.

### 4.2. Web Trading Agent

As outlined in Section 1, this section describes the internal structure of our trading agent and some details about its design and implementation. It should be emphasized that the trader agent, like all SOLERES system agents, was modeled, designed, and implemented based on run-time management of the ontologies used. The trader therefore manages two kinds of ontologies, data and functionality (or process). The first are related to the ecological information repositories which the trader can access. The second refers to trader functionality, that is, things which it can do and demand from others. In this case, behaviour and interaction protocols must be also defined. These definitions set the operating and interaction rules for agents, governing how the functions that the trader provides and demands to work (behaviour) are used and the order they are called up in (protocols or choreography). With this in mind, certain development details must be explained. Since the work perspective presented here is more focused on the searching process and how the trader was designed based on the ontologies, the explanation concentrates only on the Lookup interface (definition of the rest was similar to the following explanation).

Ontologies are written in the OWL notation and formalized as a metamodel using UML class diagrams. We also use graphs to show details of relationships between properties. Throughout the explanation, these notations are used to refer to data or functionality ontology, though all three notations represent the same thing.

**Figure 3:**
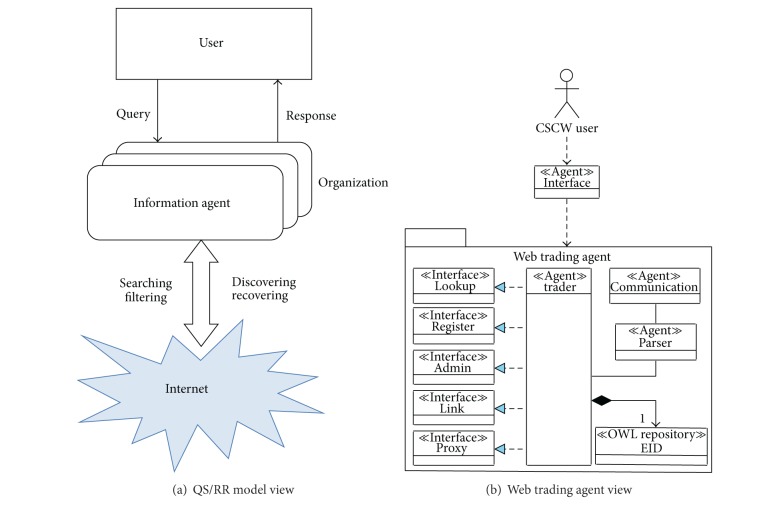
The web trading agent (b) uses the QS/RR model (a).

As mentioned above, the trading service takes part in the search for environmental information based on the QS/RR model. As shown in Figures 3(a) and 3(b), there are two basic sections in the model: (a) the user or user groups who are represented by an agent interface (Figure 3(a)) and (b) the trading service which locates the metainformation (Figure 3(b)). Regarding which it should be recalled that the SOLERES system stores (as a whole) the environmental information distributed in different OWL repositories on two levels. Some contain environmental metadata (EIM repositories) and other metadata from the first one (EID repositories). The trader manages an  EID repository.

The system makes use of ontologies. Specifically, it distinguishes data ontologies, which represent the information in the domain on two levels, and service/process ontologies, which represent the actions that can be performed in the system, the information necessary to perform them, and the results of those actions. In SOLERES, the EIM and EID data ontologies, as well as the Lookup, Register, Admin, and Link service/process ontologies, are described, and they in turn are related to the mediation system interfaces.

**Figure 4:**
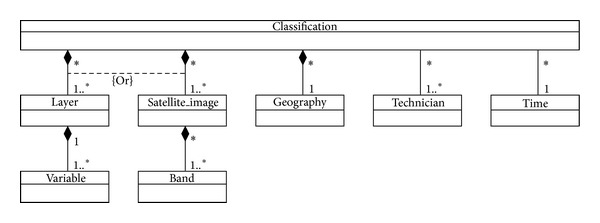
Ontology of the EID metadata that traders use.

**Figure 5:**
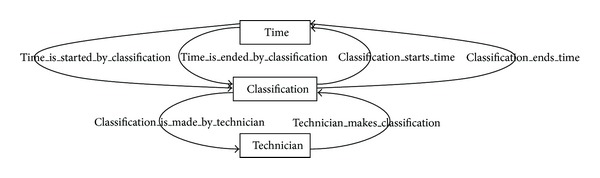
Classification is temporal information carried out by technicians.

**Figure 6:**
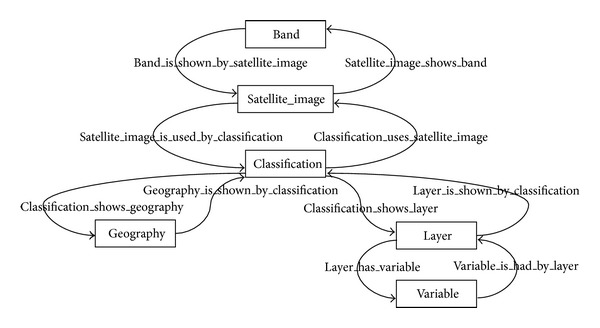
Ontological relationships of a classification.

Figure 4 shows a data ontology from an EID repository. For a better understanding, we also show two figures with the ontological relationships of the elements which describe the EID ontology (Figures 5 and 6). Let us recall that the application domain to be modeled is ecological information (a type of environmental information) on cartographic maps and satellite images. Advanced algorithms based on neuronal networks to find correlations between satellite and cartographic information were developed by the SOLERES work team. For the calculation of this correlation, prior treatment of the satellite images and maps is necessary (an image classification, Classification). A cartographic map stores its information in layers (Layer), each of which is identified by a set of variables (Variable). For instance, we are using cartographic maps classified in 4 layers (climatology, lithology, geomorphology, and soils) with over a hundred variables (e.g., scrubland surface, pasture land surface, and average rainfall).

Satellite images work almost the same way. The information is also stored in layers, but here they are called bands. An example of satellite images are the LANDSAT images, which have 7 bands (but no variables are stored in this case). Finally, both the cartographic and satellite classifications have geographic information associated (Classification), which is made at a given time (Time) by a technician or group of technicians (Technician). As a complement and formalization for graphs and metamodel, Table 2 shows the complete assertions of the eight ontology entities (the assertions may easily be interpreted from Figures 4, 5, and 6).

The functionality of our web trading agent [14, 36] is divided into three clearly differentiated components (see Figure 3): (a) a component that manages the agent-communication mechanism (Communication); (b) a parser that codes and decodes the trading ontology-based messages exchanged (Parser); and (c) trading itself (Trader). The third component is inspired by the traditional OMG trading object service specification concepts [12] (see Section 2.3). This specification indicates how offers and demands are to be implemented among objects in a distributed environment and proposes grouping all the different functionalities that a trader may include. Although the standard specifies five trader interfaces (i.e., Lookup, Register, Admin, Link, and Proxy), its specification does not demand a trader to implement these five interfaces to work. In fact, we have only developed ontologies for the  Lookup, Register, Admin, and  Link interfaces, but none has been implemented for the last one yet. The  Lookup interface offers the search-information in a repository under certain query criteria. The  Register interface enables objects in this repository to be inserted, modified, and deleted. The  Admin interface can modify the main parameters of the trader configuration, and finally, the  Link interface makes trading agent federation possible.

As previously explained, this paper focuses on identifying and explaining how ontologies appear and intervene in the web trading agent service. Of the interfaces implemented, we only explain here how the  Lookup interface works, because it takes part in the search, which is the primary subject of this paper. Several system agents, depending on their functionality, request these tasks (provided by the interfaces) from the trading agents. For example, a resource agent (belonging to a given EPU) could request the trading agent of the ambient where both are located to register a new EID document by using the  Register interface. Under these circumstances, how is this request between agents expressed? In SOLERES, this is done by specifying the service requests as ontologies. The ontologies used to describe services are known as “process ontologies,” and they are for three types of entities: concepts, actions, and predicates.

**Figure 7:**
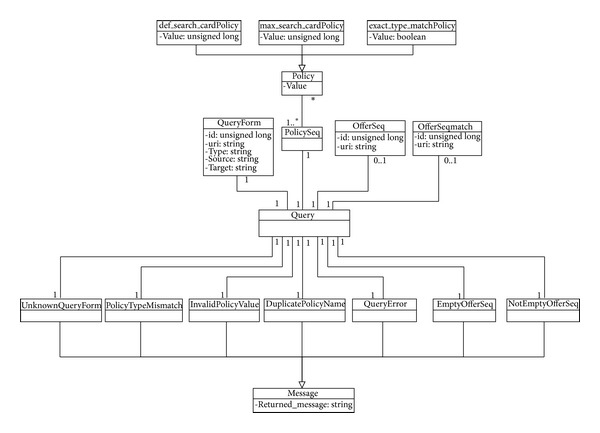
UML Lookup ontology metamodel.

In our system, an ontology has been created for each of the four interfaces (services) implemented by the trading agent. The set of all ontologies used for these trading agent services is called the trading ontology. Creation of specific ontologies for specific services allows the agents to use only the ontology that they need at any given time.  Figure 7 shows an example of a service (i.e., interface) ontology expressed in UML notation. In this case, the part of the ontology that defines the Lookup service is specified. This ontology is written in terms of concepts (domain entities), actions carried out in the domain (actions that affect concepts), and predicates (expressions related to concepts). The only action in the  Lookup service ontology is* query*, which makes the search for EID documents according to specified prerequisites. The concepts identified in the ontology are

- *QueryForm* gives the information in the EID document necessary to make the query.
- *PolicySeq* represents the set of policies that specify how the search is to be made. These policies may affect the functionality of the trader during the execution time.
- *OfferSeq* refers to the set of EID documents that meet the query requirements. It therefore represents the result of the service requested.
- *OfferSeqMach* refers to the matchmaking process result. Results can be* hard* or* soft* solutions. The first one represents an exact solution (i.e., what the user exactly wants) and the second one refers to a partial solution.

### 4.3. * OntoTrader*: Trading Models

Let us now see how the ontological web trading agent operates in the query process following the QS/RR model, since the query made by the user (or group of users) remains in the user interface until the results are retrieved.

This model is a trading-based version of the three-level client/server model. It is comprised basically of a series of objects or processes  <I,T,D>, each of which intervenes on a different level, depending on the treatment of the query. The implementation of these objects then corresponds to agents. Level 1 (L_1_) is like the client side. Queries are generated and dealt with by an interface object (I). Level 3 (L_3_) is the server side. System data (D) reside on this level. In our case, these are the EIM repositories with the environmental information. Level 2 (L_2_) is the middleware that enables the source information to be located. This is the level where the trader objects (T) operate. In our case, associated with the trader (T), the EID repositories with the source environmental information metadata (EIM) also reside there. All three objects use the Lookup ontology (defined in Figure 7) to communicate. As the premise for their functioning, an interface object must be associated with a trader object. However, a trader object can also be associated with one or more outside data sources or resources, in our case, with the environmental source data (which reside in the EPU units, as discussed above). This “trader-information source” association arises from the production of environmental information, where each EPU has an associated trader in which a subset (metadata) of environmental information generated in the EPU is registered. On the other hand, each trader can be associated with one or more traders in federations.

**Figure 8:**
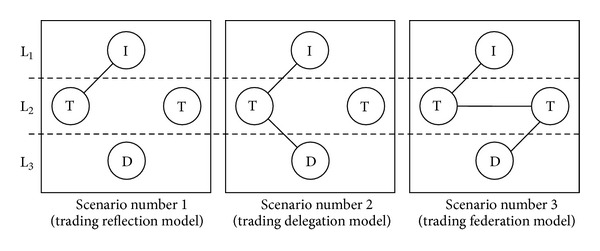
The three trading models of* OntoTrader*.

In this three-level architecture, three different scenarios or trading models are possible:* Trading Reflection*,* Trading Delegation*, and* Trading Federation*.  Figure 8 shows the three levels (L_1_, L_2_, and L_3_), where the three basic objects (I, T, and D) reside, and the three trading models are permissible in* OntoTrader*, as described below.

- The* Trading Reflection* model is a model for direct trading on the trader. The query may be solved directly by the trader. The query is generated on the interface and the information can be reached by the metadata that reside in the repository associated with the trader. In this case, the model  <I,T> pair intervenes.
- The* Trading Delegation* model trades indirectly with the trader. The query is partly resolved by the trader. A query is generated on the interface level that goes on to the trading level (T). The trader locates the source (or sources) of the source data (D) in its repository, inferring this information from its metadata repository. Therefore, the trader delegates the query to the outside data source (D). In this case, the object series intervening is  <I,T,D>.
- Finally, the* Trading Federation* model is a case in which two or more traders are able to federate. As in the cases above, the query remains preset on the interface. This query is passed on to the associated trader. The trader can propagate the query to another federated trader, who locates the external data source (D). In this case, the object series intervening is  <I,T,T,D>.

### 4.4. The Lookup Ontology in* OntoTrader*

The Lookup ontology is used between system objects. The trader uses the* Query* action and the query form concept (see the Lookup ontology, Figure 7). The  Query Form concept expresses the query in a specific language, whose properties, among others, are an  id (a query identifier) and a  uri (reference to the file where the query is stored). In addition, there could be a set of query policies (Policy) through the  Policy Seq concept, and each “policy” is represented by means of a tuple (name, value). For instance, some of the tuples implemented are def_search_card_Policy or max_search_card_Policy, indicating the number of records to be located by default and the maximum number of records to be located in the query, respectively. Possible exceptions are (Lookup ontology, Figure 7).

- UnknownQueryForm indicates that the query cannot be answered because the file specified in the  uri is not accessible.
- PolicyTypeMismatch indicates that the type of value specified is not appropriate for the  policy.
- InvalidPolicyValue indicates that the  policy value specified is not within the permissible value range for that  policy.
- DuplicatePolicyName indicates that more than one value for the  same  policy hve been specified in the  PolicySeq.
- QueryError indicates that an error has occurred during the query.

If there is no exception and the query is successfully executed, either the  EmptyOfferSeq predicate is used when no record is returned by the query or the  NotEmptyOfferSeq predicate, when some record is returned. This, in turn, uses the  OfferSeq concept to represent the set of records located in the query, whose properties are the query “id” and the file “uri” where the records found are stored.

## 5. Formalization of Ontological Web Trading

This section presents the formalization of ontological views of the* query* and* response* actions for the proposed web trader agent. Both actions are described by means of theLookup ontology (see Figure 7). We focus only on the formalization of the Lookup ontology because of its relationship with the QR/RR process.

**Definition 1 (web trader)**

A web trader *𝒯* is determined by two sets *𝒯* = {*𝒪*, *𝒢*}, where *𝒪* is a set of two types of ontologies (data and functions) *𝒪* = {*𝒪*_*d*_, *𝒪*_*f*_} and *𝒢* is a set of scheduling and control agents, where

- *𝒪*_*d*_ is an OWL repository used by the trader,
- *𝒪*_*f*_ is a set of five ontologies: *𝒪*_*f*_ = {*ℒ*, *ℰ*, *𝒟*, *𝒦*, *𝒳*},

where *ℒ* is the ontology of the Lookup interface, *ℒ* ≠ *∅*  (mandatory).*ℰ* is the ontology of the Register interface, *ℰ* ≠ *∅*  (mandatory). *𝒟* is the ontology of the Admin interface. *𝒦* is  the  ontology  of  the  Link  interface.  *𝒳* is the ontology of the Proxy interface.

In the framework of SOLERES system, ontological data *𝒪*_*d*_ are related to ecological information repositories written in OWL. Ontological functions *𝒪*_*f*_ refer to trader functionality. There are at least two ontological functions: (a) the Lookup ontology (*ℒ*), which models the query made to the trader about information (templates) stored in the trading service, and (b) the Register ontology (*ℰ*), which models the export (register) of OWL instances in the trader. The three remaining ontologies are not mandatory in a web trader. The Link ontology (*𝒟*) models the interconnection of traders (i.e., trading federation). An Admin ontology (*𝒦*) models the trader configuration (e.g., searching policies, ordering policies, and numbers of federated traders allowed). Finally, a Proxy ontology (*𝒳*) models the* legacy systems* properties in a federated trader system.

**Definition 2 (Lookup ontology)**

The Lookup interface of a web trader agent is defined as an ontology $ℒ=\{𝒞,𝒜,𝒫,ℛ,\bar{ℛ}\}$ consisting of

- *𝒞*, a finite set of concepts: $𝒞=\{ℱ,𝒴,𝒪,\hat{𝒪}\}$, where
  - *ℱ* is a query form *ℱ* = {id, uri, type, source, target};
  - *𝒴* is a policy sequence, for instance: {def_search_cardPolicy, max_search_cardPolicy, exact_type_matchPolicy};
  - *𝒪* is an offer sequence *𝒪* = {id, uri};
  - $\hat{𝒪}$ is an offer sequence match *ℳ* = {id, uri}.
- *𝒜*, an action *𝒜* = {𝒬} where 𝒬 is an input query action.
- *𝒫*, a finite set of predicates *𝒫* = {*ℳ*}, where *ℳ* is a returned message.
- *ℛ*, a relation of request.
- $\bar{ℛ}$, a relation of response.

*𝒜* represents the set of possible actions (in the Lookup ontology, only a Query, 𝒬), while *ℱ* is a concept related to a Query action, which represents the query made.

Predicates report any errors in the previous action (*UnknownQueryForm*,* PolicyTypeMismatch*,* InvalidPolicyValue*,* DuplicatePolicyName*, and* QueryError*) or a successful action; that is, it has been performed satisfactorily (*EmptyOfferSeq* and* NotEmptyOfferSeq*).

**Definition 3 (relation of request)**

Given a query form concept *ℱ*, a policy sequence concept *𝒴*, and a query action 𝒬, a relation of request is defined as *ℛ*⊆(*ℱ*×𝒬)∪(*ℱ* × 𝒬 × *𝒴*).

The purpose of the relation of request is to define the relationships that exist between concepts (set *𝒞*) and actions (set *𝒜*).

**Definition 4 (relation of response)**

Given an input query action 𝒬, a returned message predicate *ℳ*, and an offer sequence *𝒪*, a relation of request is defined as $\bar{ℛ}$, a relation of response $\bar{ℛ}\subseteq (𝒬\times ℳ)\cup (𝒬\times ℳ\times 𝒪)$ $\cup (𝒬\times ℳ\times 𝒪\times \hat{𝒪})$.

Finally, there could be relationships between “Actions” and “Concepts,” between “Predicates” and “Actions,” or even between the pair “Predicates” and “Concepts.”

**Definition 5 (ontology uses)**

Let us assume a given query 𝒬, a message *ℳ*, an offer sequence *𝒪*, and an offer sequence match $\hat{𝒪}$. The uses of the ontology are defined in these three ways:

 (a) (*Q* × *M*) → *M* = *UnknownQueryForm*
 ∨  *PolicyTypeMismatch*∨*InvalidPolicyValue*
 ∨  *DuplicatePolicyName*∨*QueryError*
 ∨  *EmptyOfferSeq*
 (b) (*Q* × *M* × *O*) → *M* = *N* *o* *t* *EmptyOfferSeq*
 (c) $(𝒬\times ℳ\times 𝒪\times \hat{𝒪})\to M=NotEmptyOfferSeq$.

## 6. Implementation and Experimental Scenarios

To evaluate the proposal, a specific environment was developed for experimentation, which is available at the website:  http://tkrs.ual.es/SKRS/. Several different* OntoTrader* trading service functionality tests can be carried out in this environment. Behind it, the objects which comprise the* OntoTrader* system have been implemented by agents using the JADE platform. Three test cases, one corresponding to each of the three possible scenarios (reflection, delegation, and federation), were prepared. Below we explain some details of* OntoTrader* implementation, the framework of experimentation and testing, and the three case studies implemented.

### 6.1. Implementation

For design reasons, the three basic* OntoTrader* model levels  <I,T,D> were implemented by agents. The interface (I) was implemented by two agents: the  Interface Agent and the  IMI Agent. The trading level (T) was implemented by another two agents: Query Agent and  Trading Agent. The data level (D) was implemented by a  Resource Agent.

The  Interface Agent is the agent in charge of receiving the queries from the user over the user interface, transferring them to the  IMI Agent for management and returning the result to the user. The  IMI Agent acts like a hub between the user interface and the rest of the modules and is responsible for managing user demands. The  Query Agents are in charge of solving the information queries demanded. The  Trading Agents enable search and location of the information in the system and a filter based on the query parameters. Finally, the  Resource Agents are responsible for managing the information bases.

**Figure 9:**
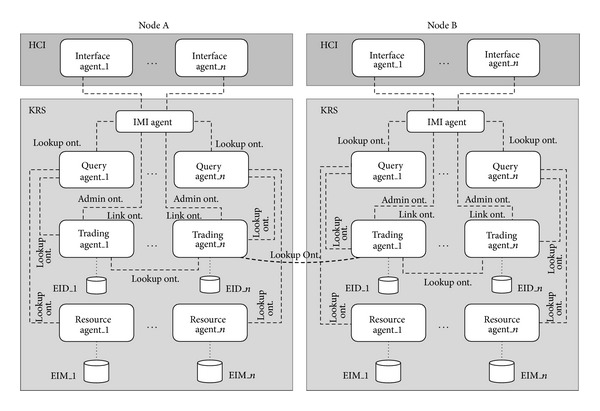
The general* OntoTrader*-based architecture.

Figure 9 shows the multiagent architecture for the Trading-Based Knowledge Representation System design under these premises. As observed in the figure, the information system may be distributed in different nodes, each of which may be comprised of a series of agents according to the configuration desired. As their basic configuration, each node must have at least one set of interface agents (made up of one or more agents), an IMI agent and a set of Query Agents (also made up of one or more agents). A node may also contain zero or more trading agents and resource agents, but the system as a whole must have at least one agent of each type in some of its nodes. Let us further recall that the trading agents have an associated repository (EID), which contains environmental information metadata. The resource agents also have associated repositories with the original data sources containing the environmental information (EIM). The dashed lines in the drawing represent the types of ontologies that intervene in agent communication. There are three basic ontologies in our system: Lookup, Admin, and Link. In this paper, we have only discussed the Lookup ontology as an example of the process ontologies. The other two are outside the scope of this study.

### 6.2. Test Scenarios

Given the agent architecture, a multitude of scenarios could be suggested for processing a query made by a user. All the possible scenarios result in a combination of the three main scenarios which we have identified and used to evaluate and validate the proposal.

- Scenario number 1 (reflection). The user query can be solved directly by a trading agent without the action of any of the resource agents.
- Scenario number 2 (delegation). The user query cannot be solved directly by a trading agent and requires the action of one or more resource agents.
- Scenario number 3 (federation). The user query can be solved by a federated trading agent or other trading agents with/without the action of a resource agent.

**Figure 10:**
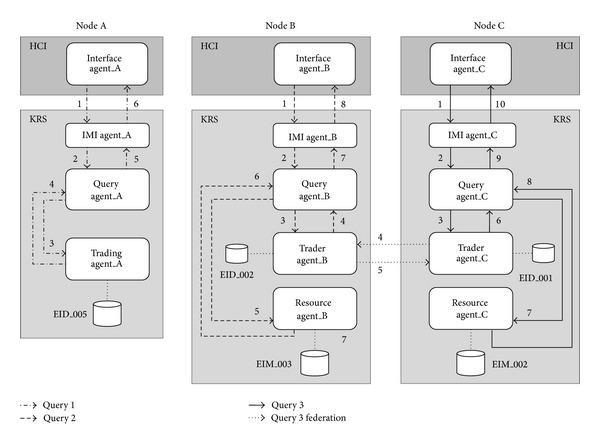
The three main scenarios (reflection, delegation, and federation).

Figure 10 shows the three basic architectures defined, where the number of agents that intervene in each scenario can be seen. The dashed lines show the order of the calls between agents. The diagram shows the QS/RR model underlying the* OntoTrader* model, which was implemented as a prototype for testing and validating the web trader agent implementation. Keep this figure in mind during the description of the three scenarios below.

In general, the sequence of the three models is very similar, as follows. The sequence begins when a user writes a query from the user interface. The following section shows how a query from the user interface is constructed. Connected to the interface is an interface agent which is in charge of translating this query into the SPARQL query language. The requests that are sent between agents are constructed based on “QUERY-REF” forms in which the query is embedded in SPARQL. The results returned by the agents are constructed using “INFORM-REF” forms. We show some of these forms further below. The interface agent transfers the query to the IMI agent which is in charge of deciding which query model to follow (reflection, delegation, and federation) based on the type of data in the query. The IMI agent tells the Query Agent which path to take. The tests for the three scenarios (three paths) are shown below.

#### 6.2.1. Scenario Number 1 (Reflection)

**Figure 11:**
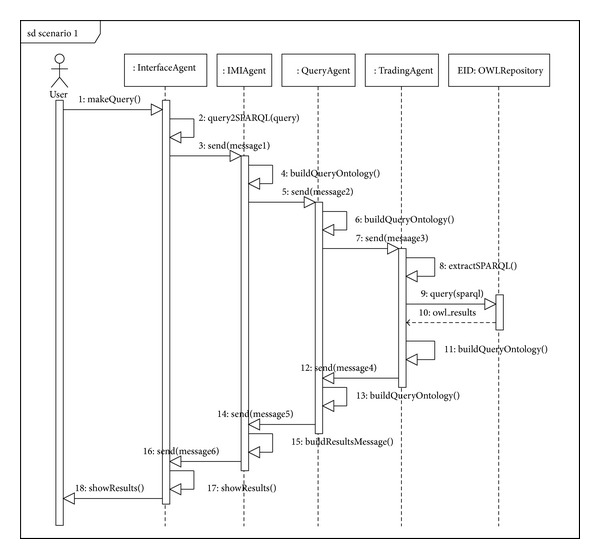
Sequence diagram for Scenario number 1 (reflection).

Figure 11 shows the sequence diagram for the reflection trading model. This scenario takes place when there is a query with the result that can be directly found from the metadata stored in the trading agent's repository. An example of a query in this scenario could be “*Find the name of the soil science classification variables for the year 2008*.” As mentioned, we show further below how a query is formed from the user interface (message number 1 in Figure 10 and sequence number 3 in Figure 11). The interface agent translates this query into SPARQL (sequence number 2 in Figure 11) and generates a  QUERY-REF message which it passes on to the IMI agent. The message form is as shown in Algorithm 1.

A QUERY-REF form always has four basic clauses: the  :sender clause (line number 2), where the name of the agent sending the message is given (in our case in the implementation, the interface agent is called  WIAgent, and it resides in the  SOLERES-KRS workspace), the  :receiver clause (line number 4) where the name of the agent that receives the message is given (in this case  IMIAgent), and the  :content clause which contains the content of the message. Observe how the query is made in SPARQL in the message content (lines number 7 to number 20). Finally, the  :language clause is where the type of language used to write the message content is given. In this case, it indicates that the content is written in SPARQL.

When the IMI agent interprets the query, it detects a query that can be solved directly by the trader (reflection model). The IMI analyzes the query sentence and the types of metadata in the trader repository to determine whether the query can be solved directly by the trader. If so, it resends this request to a Query Agent. In the case of the example above (Scenario 1), all the types of data in the query are available in the trader. The message to the Query Agent is as shown in Algorithm 2 (message number 2 in Figure 10, sequence number 5 in Figure 11).

Certain parts of the message have been omitted to reduce its length in this paper. The OWL ontology is used to write the content of this message (RDF notation, lines number 2 to number 11). The argument in the  :content clause is a document in RDF. Specifically, the Lookup ontology is used for this type of request between agents. The message makes use of the “concept/action” pair in the ontology to formulate the query. Specifically, the concept  QueryForm (lines number 2 to number 11), where the query (generated in SPARQL) and the action to be performed, Query, are specified (line number 10), is used. Observe how the concept and action were defined in the ontology in Figure 7. The previous message (Algorithm 2), as in the  :language clause, gives the type of ontology used (line number 12). The new  :ontology  clause gives the path where the ontology is found.

When the Query Agent receives this message, it infers that this query can be solved directly by the trading agent and generates a new  QUERY-REF message similar to the one before, again using the Lookup ontology (sequence number 6), and sends it to the trading agent, which launches the  sparql query to its repository (sequence number 9), obtaining results. These results are returned to the Query Agent, constructing an  INFORM-REF form in this case, as shown in Algorithm 3 (message number 4, sequence number 12).

The ontology series “concept/action/predicate” is used to construct this message. The concept  OfferSeq (lines number 4 to number 24) in the Lookup ontology shows the results found after processing the query. The action  Query (line number 26) and the predicate  NotEmptyOfferSeq (line number 25) show that the query returns a result, all of them in the Lookup ontology. If the query should not return any result, or if there were an error in the message constructed, the predicate  EmptyOfferSeq  or some predicates in the ontology indicating error would be used instead of the concept  OfferSeq, respectively.

Then, when the Query Agent receives the results from the trading agent, it transfers them directly to the IMI agent, constructing a new  INFORM-REF form with the headings  :sender and  :receiver (message number 5, sequence number 14). The IMI agent performs the same operation in turn, constructing its return form for the interface agent (message number 6, sequence number 16), which decodes the message and shows the results on the user interface, thus ending the trading reflection sequence in Scenario number 1.

#### 6.2.2. Scenario Number 2**  **(Delegation)

**Figure 12:**
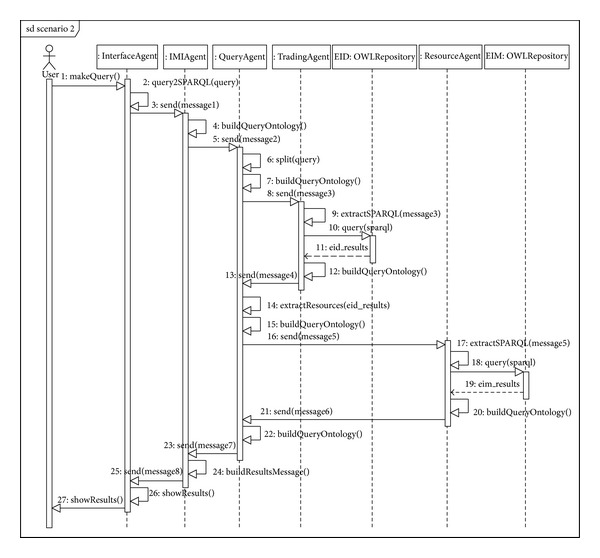
Sequence diagram for Scenario number 2 (delegation).

Figure 12 shows the sequence diagram for thedelegation trading model. In this scenario, the user query cannot be solved directly by a trading agent and requires the action of one or more resource agents to complete it. A possible query that would enter in this scenario could be* “Find the minimum value of variable X used in information layer Y.”* In this case, the result cannot be found directly in the meta-metadata that the trading agent stores in its repository and the metadata in the resource agent must be queried.

It may be observed that the interface agent, IMI agent, and Query Agent sequence are the same as for reflection (Scenario number 1). In this case, the above query generates the text in SPARQL shown in Algorithm 4, which is transmitted on  QUERY-REF  forms (for the sake of simplicity, only the SPARQL query is shown) (see Algorithm 4).

In this example, the variable X remains on the user interface as variable “E6” and layer Y is given as “Edaphic sectors.” When the form gets to Query Agent (sequence number 5 in Figure 12), it infers that the type of query is by delegation and makes a  split (sequence number 6) to prepare a double query. The first is the original query, which continues its course to the trading agent (sequence number 8). After querying its associated EID repository, the trading agent returns a message (Algorithm 5) to the Query (sequence number 13, in the sequence diagram of the Scenario 2 (see Figure 12)).

This message states that the trader has found variable “E6” in its repository in document “EIM_0000000003” coded as “VAR_0000000202”. Notice that “EIM_0000000003” is a metadata (i.e., it resides in the trader's EID repository) of an EIM resource found in a resource agent repository. This message is received by the Query Agent which generates a new message using the Lookup Ontology and applying a filter to the original SPARQL query with the data found in the last query (previous message). Specifically, the results are filtered for those documents that contain the variable called “VAR_0000000202.” This message is sent to the resource agent so it can complete the query by applying this filter. The new message (with the filter) is as shown in Algorithm 6.

Finally, the sequence ends by returning the results between agents until arriving at the interface agent, which is in charge of displaying the results on the user interface.

#### 6.2.3. Scenario Number 3 (Federation)

As mentioned, the federation-based model arises when the system has been configured for the trader to propagate the query to a second federated trader if the query could not be satisfied by the first. In this model, several different combinations are possible. One situation is that the second trader can satisfy the query directly in what is known as a case of “reflexive federation,” that is, a federation model on the first trader and in continuation reflection on the second. Another situation appears when the second trader redirects the query to a data resource (resource agent). For this case, a “*delegated federation*” appears, that is, federation on the first trader and then delegation to the resource. There could also be a case of “multiple federation” where the system has been configured for queries to be propagated among federated traders if they cannot be satisfied.

**Figure 13:**
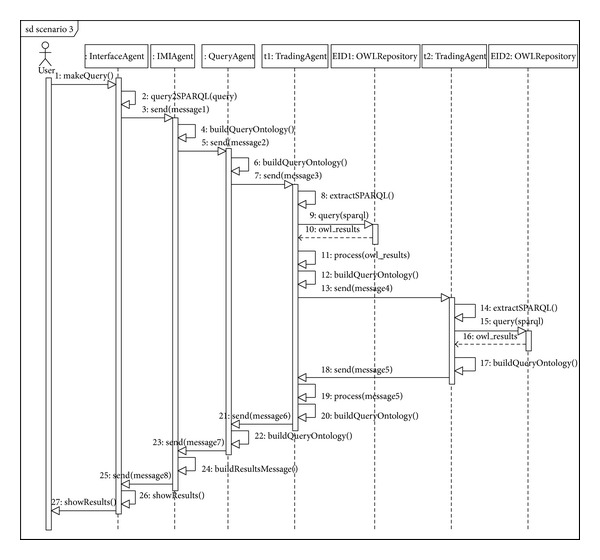
Sequence diagram for Scenario number 3 (federation).

Figure 13 shows the sequence diagram that follows the federation-based trading model. For the sake of simplification, the diagram presents a case of “reflexive federation.” The functioning for the rest of the possible combinations is similar.

One possible query in this scenario could be the following:* “Find the identifier of the classifications of city X in which there is information on variable V1 and variable V2.”* From the type of query, the data may be found in the metadata of a trading repository. For this example, we assume that Variable V1 is a metadatum in the repository of one trader, and Variable 2 is in the repository of another.

In the example of the query above, the following sentence is generated in SPARQL. The sequence of the  QUERY-REF calls and their corresponding  INFORM-REF among the system agents is similar to those explained in the scenarios above (see Algorithm 7).

### 6.3. Experimentation Environment

**Figure 14:**
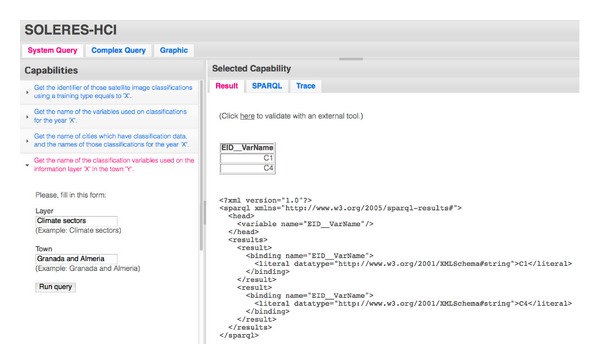
A part of the  System Query window in the test environment.

As mentioned, to evaluate the proposal, a specific experimental prototype was developed for testing. The three scenarios posed in the section above can be put into practice at the test website  http://tkrs.ual.es/SKRS/.  Figure 14 shows a portion of the window of this test scenario. This website consists of three basic sections: the “System Query,” which contains a series of system capabilities with a basic list of preset queries, the “Complex Query” section, where any type of complex query can be constructed, and the “Graphic” section, where the information repositories used can be displayed graphically as a tree.

The preset queries section, “System Query,” is divided in half vertically into two zones (Figure 14). On the left, the user has a list of capabilities that represent basic system queries. When one of them is selected, a menu drops down with the parameters that must be entered before it can be executed. In the example in the figure, the user wants to know the names of the classification variables used in the information layer, “Climate sectors” in the cities of “Granada and Almeria”.

**Figure 15:**
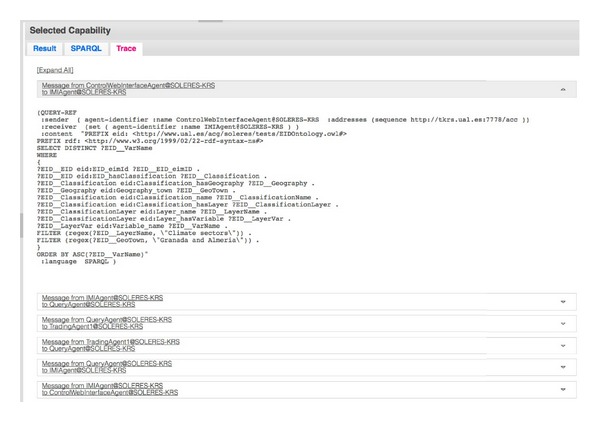
Trace of messages sent between agents after the query.

After executing the query, in the right-hand part of the window, certain information appears which is organized in turn in other three sections. On one hand, the result of the query is shown in XML format (“Result” tab). From this zone, it can be checked whether the result returned by the system is well constructed by making use of a standard external (third-party) tool, which does not belong to the system, called “Pellet.” Another capability enables the query made to be displayed in SPARQL. In order to validate the implementation, we have included a third capability which makes it possible to trace the messages exchanged by the different system agents. The traces of the messages exchanged among system agents after executing the query above are shown in the “Trace” tab (see Figure 15). These traces can be folded up or dropped down to facilitate supervision and checking of the messages generated in the query sequence. The figure shows the  QUERY-REF message, which was generated and transmitted from the interface agent to the IMI agent. It is also possible to observe the SPARQL sentence generated in this message.

**Figure 16:**
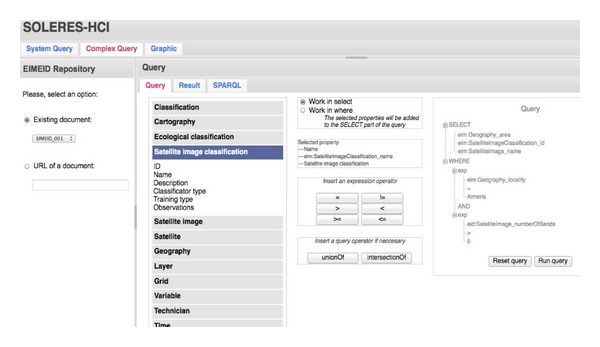
A portion of the  Complex Query window in the test environment.

Any type of complex system query can be constructed in the section “Complex Query.” This section is also divided vertically into two parts (Figure 16). On the left, the test repository (on which the complex query will be executed) can be selected. Any EIM or EID repository in the test environment can be selected, or else the URL where the repository is found can be specified. On the right, the query can be constructed from the “Query” tab, by selecting the fields and criteria for the query and specifying their values. When constructing the query, the attributes to be selected can be incorporated in either the SELECT or the WHERE part of the query. All the properties selected are incorporated in the SELECT part of the query. When the option marked is related to the WHERE part of the query and one of the attributes in the list on the left has been selected, the option to select one of the operators for filtering the condition to be set appears on the query interface. When the operator has been selected, it is added to the tree on the right where the query is saved and then the value to be checked must be entered for the WHERE clause filter. Additional  insertionOf (AND) and/or  unionOf (OR) type conditions may also be added. Once the query is executed, the result of its execution appears on the “Result” sheet, as shown in XML.

The section “Graphics” of the website enables graphical display of the information repositories. A basic repository of the system may be displayed, or it is also possible to introduce a URL where an external repository is located. On the right-hand side of the window, an “applet” draws its content graphically. This “applet” can also make searches for concepts in the data ontology represented.

## 7. Evaluation and Validation:**  **Final Considerations

**Figure 17:**
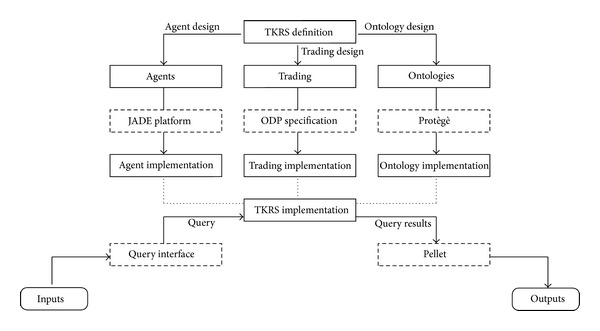
*OntoTrader* restrictions for TKRS validation.

For the validation of our environmental information system, the starting point is establishing certain “contracts” or restrictions to be evaluated in different areas of our framework.  Figure 17 shows a summary of the steps in developing the TKRS (Trading-based Knowledge Representation System). The three main features of the TKRS are trading, software agents, and the ontologies.

Each of these features has a series of restrictions that make it possible for the system implemented to function as desired (validation). In the case of the trading function, a trader was designed and implemented following the standard RM-ODP specification, which enabled us to develop a trading system with the functions we required properly. The ODP trading standard was described in Section 2.3.

Several different system objects (including the trader) were implemented as software agents using the JADE platform, which enabled us to validate their behavior, as well as their proper functioning. Furthermore, JADE is an implementation framework that complies with the FIPA (FIPA,  http://www.fipa.org/) (*Foundation for Intelligent Physical Agents*) specification. FIPA is an IEEE Computer Society Standards organization that promotes agent-based technology and the interoperability of its standards with other technologies.

Besides the trading and software agents mentioned above, the third key feature in the development of the TKRS is the ontologies (see Section 4 for further details). Their design is assumed to be correct for two reasons: (1) the process ontologies were developed with the JADE specification for messages exchanged by agents and under the trader ODP interface specification and (2) the data ontologies were found from questionnaires filled out by the experts who made the data classification in the framework of the national project this research a part of [4]. Based on this design premise, it was implemented using the Protégé (Protégé,  http://protege.stanford.edu/) tool, a standard ontologies tool, and with this tool we were able to verify that the implementation of the ontologies is correct.

When the TKRS system had been implemented, a series of “contracts” or restrictions were placed on data input and output (Figure 17). The restrictions on input enabled us to verify that the queries executed in the system are properly constructed queries that can be translated correctly into SPARQL and can be correctly launched in TKRS. These restrictions were made using a query interface which guided us in constructing executable system data queries. This can be checked on the website test environment specially developed for validating and evaluating the proposal:  http://tkrs.ual.es/SKRS/.

Finally, once the results of the queries have been returned by the TKRS, another feature of the system is in charge of checking that the data obtained are correct. This feature is an external check (Pellet) to which our data ontology and the SPARQL query made are sent as parameters, obtaining as a result the data that must be returned to the query. It also checks the validity of the ontology, warning if there is any type of error in it. TKRS implementation was checked with these five system features (marked in Figure 17 with a double box), and the functioning of the QS/RR data recovery model proposed was validated.

Furthermore, several sample scenarios were developed where the different types of query that can be executed were tested. In addition, a tool with which several evaluation and validation tests, predefined system queries, generic queries on system documents, display of the system document ontologies, and so forth, can be made was developed and is available on the web. The tool has a mechanism for agent message* traceability* and can check and validate their functioning and proper implementation. The link to this tool is included in the paper, so anyone can make the pertinent validation and evaluation tests. For the time being, these information retrieval (IR) evaluation techniques only examine whether information retrieval is done correctly and that the data are what was asked for.

## 8. Conclusions and Future Work

Modern information systems are increasingly required to provide support for users who are in different places and have different types of data, facilitating access to information, decision-making, workgroups, and so forth.* Distributed Information Systems* (DIS) appear to provide the answer to these new requirements [37]. Web-based Information Systems (WIS) [2], for instance, are developed under open, distributed paradigms. This involves the use of rules and standards for their construction and real time operation, interaction, and interconnection. In this kind of system, interactions are between system “agents” (e.g., web components, subsystems, and humans) working in the same ambient (computing space), or even with other third-party “agents.” In both cases, the knowledge semantics for managing each part (i.e., software agent in our case) of the system must be formally defined [38].

EMIS, a type of WIS, have been under development in the last few years [3]. The EMIS are social and technical systems with a variety of final users and actors (i.e., politicians, technicians, and administrators) who cooperate with each other and interact with the system for decision making, problem solving, and so forth. Today, web-based EMIS greatly facilitate information search and retrieval, favoring user cooperation and decision making. Their design requires the use of standardized methods and techniques that provide a common vocabulary to represent the knowledge in the system and a capability for mediation to allow interaction (communication, negotiation, coordination, etc.) of its components. Ontologies are able to provide that shared vocabulary, and trading systems can improve the interoperability of open, distributed system components.

The present paper showed how traditional traders, properly extended to operate in WIS, are a good solution for information retrieval. An example of web-based EMIS is the SOLERES system, a spatiotemporal environmental management system based on neural networks, agents, and software components [4]. In this paper, we have reviewed the most important EMIS in the literature and have compared them, especially their agent, trader, and ontology features. We have also introduced ontological web trading (*OntoTrader*), an extension of the traditional OMG trading service to support ontological information retrieval issues on web-based EMIS. The paper also presents three trading models for “information retrieval”: trading reflection, trading delegation, and trading federation.

We have also shown an* OntoTrader* model implementation using software agent-based approaches. This kind of web service is a search service based on a “Query-Searching/Recovering-Response” web model using the SPARQL query language and the* OntoTrader* ontology description language for information retrieval. This service is based on a user request action that identifies the agents involved and their communication protocols. Ontologies are used in two different contexts: (a) to represent the application domain information itself (data ontology) and (b) the services that some agents request from others during their interaction (process ontology). In this paper, we have described the process and data ontology design features of the Lookup trader interface. All research work presented in this paper was part of the complete *Ontology-Driven Software Engineering* (ODSE) design strategy we are now developing in SOLERES. Implementation details and a prototype of* OntoTrader* are available at  http://tkrs.ual.es/SKRS/.

Future work will focus on several open lines of research. On one hand, we are studying the possibility of grouping all ontology management under a single agent (ontology agent). This agent would manage a database with all the ontologies used in the system and would code/decode them. This would considerably simplify the implementation of other agents and would provide more efficient management. Our work regarding the implementation of SOLERES-HCI (human-computer interaction) is also ongoing. This level of the EMIS is defined by means of the Computer Supported Cooperative Work (CSCW) paradigm [39] and implemented by using an innovative technology of intelligent agents and multiagent architectures. Furthermore, we continue working on this subsystem, which is described throughout the paper, and we are studying how to decompose the user tasks into actions that will have to be performed by the SOLERES-KRS subsystem for retrieval of the information requested and the ontology mapping problems involved.

In literature review, there are no approaches with enough similarities to make an empirical comparison between our processes and other existing algorithms. However, we intend to add some experimental comparisons with some isolated parts of other works, in order to highlight the advantages of our proposal. Finally, we would like to study, develop, and incorporate new evaluation and validation techniques, such as measuring the precision of data returned to queries, response time in executing the query, and usability.

## Figures and Tables

**Algorithm 1:**
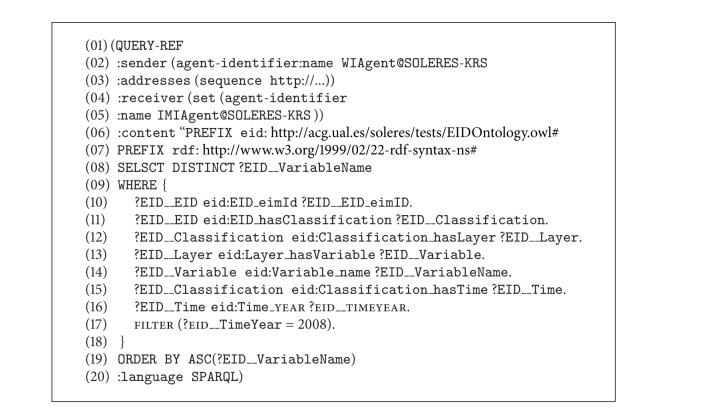


**Algorithm 2:**
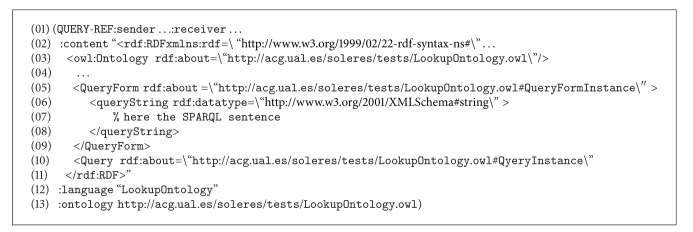


**Algorithm 3:**
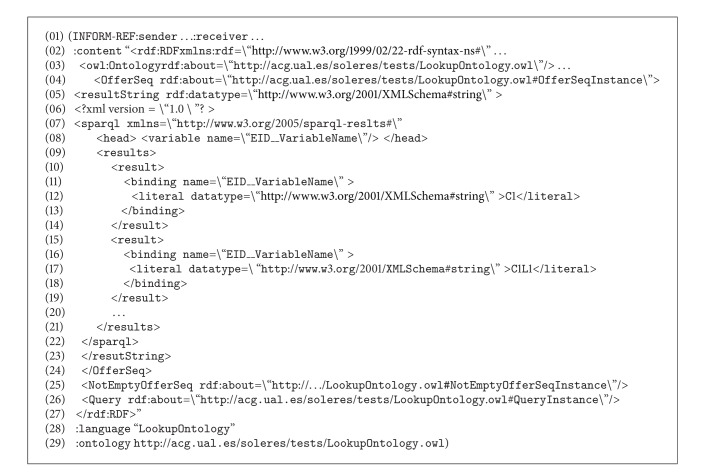


**Algorithm 4:**
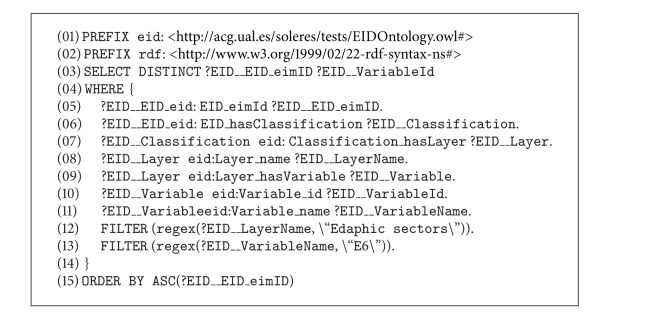


**Algorithm 5:**
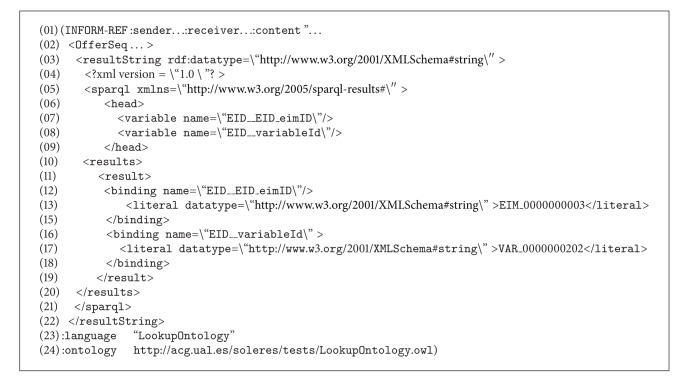


**Algorithm 6:**
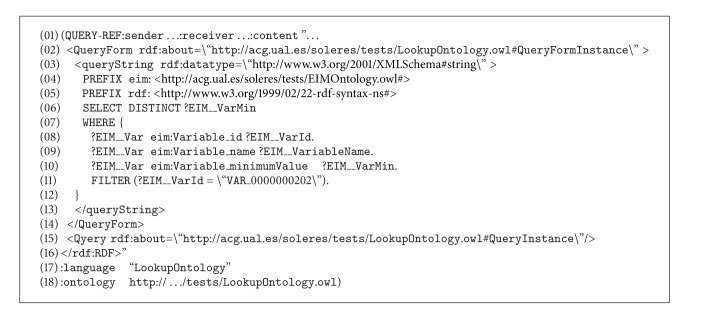


**Algorithm 7:**
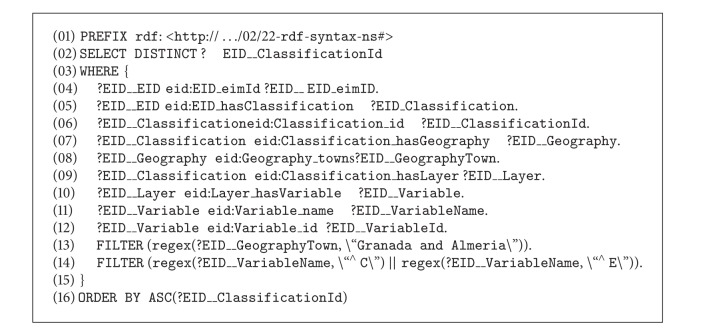


**Table 1:** A comparative view of some environmental management system architectures.

System	Trading	Ontologies	User agent	Technology	Application domain
InfoSleuth	No	Yes	Yes	XML/RDF, KQML, and OKBC	Water resources
EDEN-IW	No	Yes	Yes	JADE, DAML-OIL	Water resources
NZDIS	No	No	Yes	CORBA/OQL, MOF	Environmental data
FSEP	No	No	No	JACK	Meteorology
MAGIC	No	No	Yes	FIPA-ACL, CORBA	Water treatments
DIAMON	No	No	Yes	Java/C++, FIPA-ACL	Water treatments
BUSTER	Yes	Yes	No	OIL, FIPA-OS	Geographical information

SOLERES	Yes	Yes	Yes	JADE, OWL, SPARQL, and UML	Ecology

**Table 2:** Formal EID ontology assertions.

Number	Entity	Assertions
Number 1	Band	(band_id exactly 1) and (band_is_shown_by_satellite_image min 0) and (band_name exactly 1)

Number 2	Classification	(classification_id exactly 1) and ((classification_shows_layer min 1) or (classification_uses_satellite_image min 1)) and (classification_ends_time exactly 1) and (classification_is_made_by_technician min 1) and (classification_name exactly 1) and (classification_shows_geography exactly 1) and (classification_starts_time exactly 1)

Number 3	Geography	(geography_id exactly 1) and (geography_is_shown_by_classification min 0) and (geography_locality exactly 1) and (geography_name exactly 1) and (geography_town exactly 1)

#4	Layer	(layer_id exactly 1) and (layer_has_variable min 1) and (layer_is_shown_by_classification exactly 1) and (layer_name exactly 1) and (layer_observations max 1)

Number 5	Satellite_image	(satellite_image_id exactly 1) and (satellite_image_is_used_by_classification min 0) and (satellite_image_shows_band min 1)

Number 6	Technician	(technician_id exactly 1) and (technician_first_name exactly 1) and (technician_last_name exactly 1) and (technician_makes_classification min 0) and (technician_organization max 1)

Number 7	Time	(time_id exactly 1) and (time_day exactly 1) and (time_month exactly 1) and (time_year exactly 1) and (time_is_started_by_classification min 0)

Number 8	Variable	(variable_id exactly 1) and (variable_name exactly 1) and (variable_is_had_by_layer exactly 1)
